# Periodontal status, perceived stress, diabetes mellitus and oral hygiene care on quality of life: a structural equation modelling analysis

**DOI:** 10.1186/s12903-020-01219-y

**Published:** 2020-08-20

**Authors:** Vanessa Machado, João Botelho, Luís Proença, Ricardo Alves, Maria João Oliveira, Luís Amaro, Artur Águas, José João Mendes

**Affiliations:** 1 Periodontology Department, Clinical Research Unit (CRU), Centro de Investigação Interdisciplinar Egas Moniz (CiiEM), Instituto Universitário Egas Moniz (IUEM), Egas Moniz Cooperativa de Ensino Superior Campus Universitário, Quinta da Granja, 2829 - 511 Almada, Portugal; 2 Quantitative Methods for Health Research (MQIS), CiiEM, IUEM, Almada, Portugal; 3 Department of Anatomy and Unit for Multidisciplinary Research in Biomedicine (UMIB), Institute of Biomedical Sciences Abel Salazar (ICBAS), University of Porto, Porto, Portugal; 4 Health Centers grouping (HCG) Almada-Seixal, Regional Health Administration of Lisbon and Tagus Valley (RHALTV), Lisbon, Portugal; 5 Clinical Research Unit (CRU), CiiEM, IUEM, Almada, Portugal

**Keywords:** Andersen’s behavioral model, Periodontal diseases, Periodontitis, Self-perceived stress, Oral health behaviors, Oral health-related to quality of life

## Abstract

**Background:**

To determine if periodontal risk assessment (PRA), the number of missing teeth, diabetes mellitus (DM), perceived stress and interproximal cleaning are associated with oral health-related quality of life (OHRQoL), using Andersen’s behavioral modelling (ABM).

**Material and methods:**

Data derived from 472 adults derived from a representative population of the Study of Periodontal Health in Almada-Seixal (SoPHiAS) was used. Socioeconomic status, perceived stress scale (PSS-10), oral health behaviors and oral health impact profile (OHIP-14) were collected through questionnaire. Periodontal conditions were assessed with a full-mouth periodontal examination. PRA was computed through behavioral and clinical information. Variables were grouped into Predisposing Factors, Enabling, Need, Oral Health Behaviors and Perceived Health Outcome latent variables. Confirmatory factor analysis, structural ABM and model fitness were conducted.

**Results:**

ABM applied to OHIP-14 showed acceptable model fit (χ^2^ = 2.75, CFI = 0.92, TLI = 0.90, RMSEA = 0.05, CI 90% [0.04–0.07]). The average of OHRQoL was 9.5 ± 11.3. Patient with periodontitis and with a high number of missing teeth experienced worse OHRQoL. Uncontrolled DM participants had more periodontal treatment necessity and poorer OHRQoL. Characteristic like aging and lower levels of education were directly associated with better OHRQoL, but in indirect path the OHRQoL was diminishes. Good oral hygiene and preventative measures were associated to lower periodontal treatment necessity. Lower periodontal treatment necessity was associated to higher OHRQoL. Age, tooth loss and interproximal cleaning were the most associated items to Predisposing, Need and Oral Health Behaviors, respectively.

**Conclusion:**

ABM confirmed age, number of missing teeth, DM, interproximal cleaning and perceived stress as associated factors for OHRQoL. Uncontrolled DM was associated to higher Need and poorer OHRQoL. Good oral hygiene habits promote a healthy periodontium and, consequently, increases OHRQoL.

## Introduction

Periodontal diseases (PD) are one of the major global public health problems [1]. Globally, adult populations suffer from mild to moderate periodontitis, while severe periodontitis prevalence range from 5 to 20% [2–9]. Consequently, the economic burden of PDs was estimated to be profoundly impactful globally, with over fifty billion dollars in indirect costs due to severe periodontitis [10, 11].

Over the past decades, several risk factors have been implicated in the onset and progression of PD such as age, gender, socioeconomic status, low education levels [12–15], diabetes mellitus (DM) [16], smoking and oral hygiene habits [17–20] and psychosocial factors, in particular stress [21, 22]. Thereupon, the impact of PD on oral health-related quality of life (OHRQoL) became an important research matter. Many lines of evidence have proven that the worsening and extent of PD is very deleterious towards OHRQoL [23–27], though the treatment of PD can restore good OHRQoL levels [28]. Also, lifestyle habits and awareness towards periodontitis are strongly related to oral health behaviors [29]. Therefore, and considering the complexity of factors related to PD, the implementation of holistic periodontal risk network analyses has been gaining preponderance.

Currently, structural equation modelling (SEM) is a very popular strategy to investigate direct and indirect associations between several contributing factors [30]. Previously, SEM has been employed to assess the relationship of PD with anxiety and depression [31], fear of pain, dental fear and OHRQoL [32, 33], and chronic systemic diseases [34–36].

**Fig. 1:**
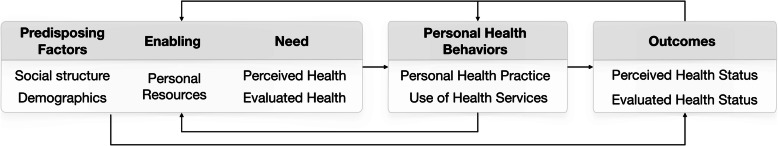
Model of health services’ use and health outcomes based on Andersen’s behavioural model (1995)

One of the best known SEM approaches is Andersen’s behavioral modelling (ABM), used to investigate the factors that interfere with the access to medical care [37] (Fig. 1). In detail, ABM was initially developed to offer a scientific understanding under a complex structure including health outcomes and their social, behavioural and attitudinal determinants towards the use of health services [37]. In a subsequent investigations, ABM has been employed in dental care and oral health outcomes using the cost of treatment and key psychosocial factors [33, 38, 39], revealing a particular importance for OHRQoL [33, 38]. Nevertheless, no study has introduced other relevant variables in an ABM approach in adults, such as the number of missing teeth, Periodontal Risk Assessment (PRA), periodontal diagnosis according to American Academy of Periodontology (AAP)/European Federation of Periodontology (EFP), DM, interproximal cleaning and self-perceived stress.

Therefore, we aimed to investigate whether the number of missing teeth, PRA, DM, interproximal cleaning and self-perceived stress are relevant factors towards OHRQoL through ABM, in the adult population of the Study of Periodontal Health in Almada-Seixal (SoPHiAS) survey.

## Materials and methods

### Ethics and study design

The SoPHiAS is a cross-sectional representative study in the municipalities of Almada-Seixal, Portugal [12]. This study was approved by the Research Ethics Committee of the Regional Health Administration of Lisbon and Tagus Valley, IP (Portugal) (Approval numbers: Process 3525/CES/2018 and 8696/CES/2018) [12]. Informed consent was written obtained from all participants prior to commencement. This survey followed the STrengthening the Reporting of OBservational studies in Epidemiology (STROBE) guidelines [40].

### Setting

#### Sample size estimation and measurement reproducibility

The sampling strategy and measurement reproducibility is available in Botelho and Machado el al [12] The estimated minimum sample size for the periodontitis prevalence in the Portuguese adult population, with a margin of error of 3.0%, for a 95% confidence level, was 412 individuals, based on the previously reported national prevalence data of 10.8% [41]. The required sample was stratified according to the number of adult (age group from 18 to 64 years) subjects assigned to each Family Health Units (FHU).

For the periodontal diagnosis, measures were performed by two trained and calibrated examiners (V.M. and J.B.). The inter-examiner correlation coefficients were 0.98 and 0.99, for clinical attachment loss (CAL) and periodontal pocket depth (PPD), respectively. The intra-examiner ICC ranged from 0.97 to 0.99, for both PD and CAL.

#### Periodontal examination

We performed a full-mouth circumferential periodontal inspection with a manual periodontal North Carolina probe (Hu-Friedy® Manufacturing Inc.) at six sites per tooth (mesiobuccal, buccal, distobuccal, mesiolingual, lingual and distolingual). Thrid molars and implants were excluded from the analysis. PPD was measured as the distance from the free gingival margin to the bottom of the pocket and gingival recession (Rec) as the distance from the cementoenamel junction (CEJ) to the free gingival margin, and this assessment was assigned a negative sign if the gingival margin was located coronally to the CEJ. CAL was calculated as the algebraic sum of Rec and PPD measurements for each site. Bleeding on probing (BoP) was used to evaluated the clinical periodontal inflammation and stability [42]. No radiographic examination was performed.

Gingivitis cases were defined according to Trombelli et al. [43] and periodontitis disease severity and extent according to Tonetti et al. [44]. At the end of the examination, participants were informed about their periodontal status. Patients diagnosed with periodontal disease were referred to the Egas Moniz Dental Clinic (EMDC) for its treatment without additional costs.

### Participants

The participants of this study derive from SoPHiAS study. The exclusion criteria were participants: edentulous and 65 years old or older. From a total of 1064 subjects, a subset of 472 adults were included.

### Selection of variables

The five proposed latent variables were selected according to ABM [37] and we take into consideration three previous studies [33, 38, 39]. We included in the analysis: 1) Predisposing Factors; 2) Enabling; 3) Need; 4) Oral Health Behaviors; and 5) Perceived Health Outcome.

#### Predisposing factors

Among the predisposing factors, age educational level, occupation, and marital status constituted the social structure elements. Age was evaluated as a continuous variable. Education was categorized according to the 2011 International Standard Classification of Education (ISCED-2011) [45], and were coded as: Elementary (ISCED 0–1 levels) = 1, Lower secondary education to Doctoral or equivalent level (ISCED 2–8 levels) = 0. Occupation of each participant was classified as: student (code = 0), employed (code = 1), unemployed (code = 2) or retired (code = 3). Marital status was defined as: single (code = 0), married/union of fact (code = 1), divorced (code = 2) or widowed (code = 3).

#### Enabling

We included household monthly income (in euros), and the Portuguese version of the Perceived Stress Scale (PSS) as two items: positive factor and negative factor [46]. The PSS-10 was a 10-item tool that assesses self-perceived stress [46]. Each item was rated on a 5-point Likert scale (coded never = 0, almost ever = 1, sometimes = 2, fairly often = 3 and very often = 4). The PSS-10 was divided in two domains: six positive (items 1, 2, 3, 6, 9 and 10) and four negative (items 4, 5, 7 and 8, that require reversion) worded items.

#### Need

Need were represented by the number of missing teeth; PRA (coded low risk = 0; moderate risk = 1; higher risk =2) [47]; periodontitis extent (coded non-periodontitis = 0; localized periodontitis [< 30% of teeth involved] = 1; generalized periodontitis [≥30% of teeth involved] = 2) [44]; periodontitis staging (coded no-periodontitis = 0; gingivitis = 1; mild [Stage 1] = 2, moderate [Stage 2] = 3, and severe [Stage 3 and Stage 4] = 4) [43, 44]; BoP [42]; denture stability (coded no denture = 0; stable denture = 1; unstable denture = 2); and DM was confirmed using medical records and through the hemoglobin A1c (HbA1c) (coded according to WHO criteria [48]: non-DM = 0; controlled DM (HbA1c < 6.5) = 1; uncontrolled DM (HbA1c ≥ 6.5) = 2).

#### Oral health behaviors

The participants’ oral health behavior determinants and use of dental services were measured with the frequency of toothbrushing, used of interproximal cleaning and last dental attendance. For toothbrushing habits, we questioned “How often do you clean your teeth a day?” (coded one or less a day = 0, twice a day = 1, and more than twice a day = 2). For interproximal cleaning, we questioned “Do you regularly perform flossing or interdental brushing?” (coded no = 0, occasionally = 1, yes = 2). Dental attendance orientation was assessed in response to “When was your last visit to the dentist?” (coded more than 12 months = 0, 6 to 12 months = 1, less than 6 months = 2).

#### Perceived health outcome

OHRQoL was measured using the short-form oral health impact profile (OHIP-14) validated for Portuguese [49]. OHIP-14 assess fourteen items, each of the items rated on a 5-point ordinal scale (never = 0, hardly ever = 1, occasionally = 2, fairly often = 3 and very often = 4) [50]. As previously divided for SEM analysis [33, 38], OHRQoL was set in three major indicators – physical (items 1, 2, 3, 4, 5 and 10 were summed), psychological (items 6, 7, 8 and 9 were summed) and social impacts (items 11, 12, 13 and 14 were summed).

### Data analysis

Data were analysed using the IBM® SPSS® Statistics, v. 24 and AMOS 24. We started by performing an exploratory factor analysis (EFA) to reveal the underlying structure of the variables. Second, we performed a Confirmatory Factor Analysis (CFA) to identify the acceptability of the indicators within each latent construct [30]. CFA confirmed the scale items (indicators) representing each of the five constructs (Table 1 and Fig. 3).

**Table 1:** Characteristics of the study variables (*n* = 472)

	**Value**
**Predisposing Factors**	
Age, mean (SD)	46.1 (12.5)
Gender, n (%)	
Male	175 (37.1)
Female	297 (62.9)
*Social structure*	
Education, n (%)	
Primary school	78 (16.5)
Middle	308 (65.3)
Higher	86 (18.2)
Occupation, n (%)	
Student	19 (4.0)
Employed	284 (60.2)
Unemployed	127 (26.9)
Retired	42 (8.9)
Marital status, n (%)	
Single	145 (30.7)
Married / Union of fact	262 (55.5)
Divorced	56 (11.9)
Widowed	9 (1.9)
**Enabling**	
Household monthly income, mean (SD) (€)	1110.3 (790.6)
PSS 10 positive factor, mean (SD)	9.2 (6.0)
PSS 10 negative factor, mean (SD)	5.9 (3.3)
**Treatment Need**	
Missing teeth, mean (SD)	5.6 (5.5)
Periodontal risk assessment, n (%)	
Low	284 (60.2)
Moderate	42 (8.9)
Higher	146 (30.9)
Stages of periodontitis, n (%)	
No-periodontal disease	207 (43.9)
Gingivitis	48 (10.2)
Mild (Stage 1)	62 (13.1)
Moderate (Stage 2)	80 (16.9)
Severe (Stage 3 and 4)	75 (15.9)
Periodontitis extent, n (%)	
Localized Periodontitis	105 (22.2)
Generalized Periodontitis	112 (23.7)
Bleeding on probing (%), mean (SD)	14.0 (19.0)
Diabetes Mellitus, n (%)	
No	431 (91.3)
Yes and Hbc1A < 6.5	9 (1.9)
Yes and Hbc1A ≥ 6.5	32 (6.8)
Denture stability, n (%)	
Subjects without denture	373 (79.4)
Subjects with stable denture	87 (18.4)
Subjects with unstable denture	10 (2.1)
**Personal health practice / use of dental services**	
Tooth brushing, n (%)	
One or less a day	114 (24.2)
Twice a day	274 (58.1)
More than twice a day	84 (17.8)
Interproximal cleaning, n (%)	
Yes	106 (22.5)
Occasionally	64 (13.6)
No	302 (64.0)
Last dental attendance, n (%)	
< 6 months	140 (29.7)
6–12 months	67 (14.2)
> 12 months	265 (56.1)
**Perceived oral outcome**	
*Oral health impact profile (self-reported)*, mean (SD)	
OHIP-14	9.5 (11.3)
OHIP −14 Physical	5.7 (5.8)
OHIP −14 Psychological	2.6 (4.0)
OHIP −14 Social	1.2 (2.8)

Next, we employed a SEM analysis following an ABM procedure. In accordance with the model and following [33, 38], it was hypothesized that: ‘predisposing factors’ would predict ‘enabling’ and ‘oral health behaviors’; both ‘predisposing’ and ‘enabling’ resources would predict ‘need’ and ‘oral health behaviors’; ‘predisposing factors’, ‘enabling’ and ‘oral health behaviors’ would predict ‘need’ which would, in turn, predict ‘perceived health outcome’. In addition, ‘predisposing factors’, ‘enabling’ and ‘oral health behaviors’ would predict ‘perceived health outcome’. AMOS estimates the total effects, which are made up of both the direct effects (a path direct from one variable to another, e.g. predisposing factors → enabling) and indirect effects (a path mediated through other variables, e.g. predisposing factors → need via enabling). Given the presence of both non-normal and categorical data, the model was estimated using bootstrapping (*n* = 900+) [38]. The ML bootstrap estimates and standard errors (together with bias-corrected 90% confidence intervals [CI]) were then compared with the results from the original sample to assess the stability of parameters and test statistics [51].

As recommended [51, 52], model fit was evaluated using a range of indices from three fit classes: absolute, parsimony adjusted and comparative. We considered as an acceptable model fit if: χ2/degrees of freedom (df) ratio < 3.0; Root Mean Square of Approximation (RMSEA) value < 0.06; Confirmatory Fit Index (CFI) and Tucker Lewis index (TLI) ≥ 0.9; and a Standardized Root Mean Square Residual (SRMR) < 0.08 [52–54].

## Results

### Study sample

All participants were recruited between December 2018 and April 2019 data. Overall, 472 participants from 18 to 64 years old were included, being mainly females (62.9% vs 37.1%), middle age (46.1 ± 12.5), presenting middle education levels (65.3%), and with low prevalence of DM (8.7%). The prevalence of periodontitis was 45.9%, of which 23.7% had generalized periodontitis and 15.9% had severe periodontitis. Indeed, the mean number of missing teeth was 5.6, and 30.9% of subjects showed a high-PRA risk. Indeed, only 20.5% had denture, of which 2.1% were unstable. Mean ± SD of OHIP-14 measured were 9.5 ± 11.3. Scale items representing each of the five constructs are detailed in Table 1.

### Confirmatory factor analysis

**Fig. 2:**
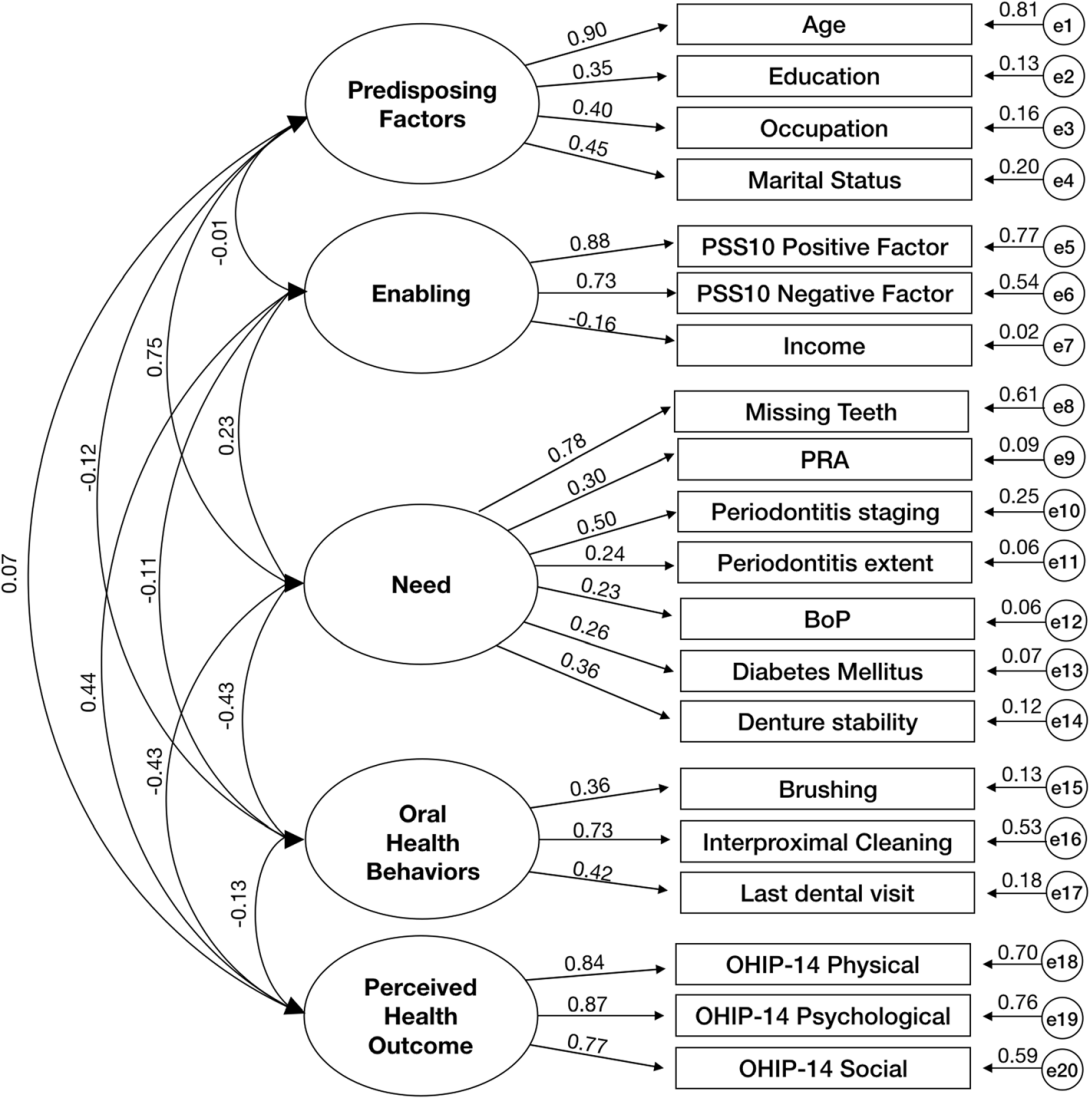
Bootstrapped ML standardized estimates for the Confirmatory Factor Analysis (CFA). All obtained effects were significant (*p* < 0.001). Factors (latent variables) are in ellipses, items (indicator variables) in rectangles and residual error terms in circles

The measurement model was an acceptable fit on three of the a priori indices (Table 2, Model 1). The correlation values within five latent variables ranged − 0.43 and 0.75, exhibiting acceptable discriminant validity (i.e. < 0.85) [51]. The bootstrapped standardized estimates for this five-factor measurement model can be seen in Fig. 2.

**Table 2:** Fit indices for the measurement and structural models

Model	χ^2^/d.f.	p	RMSEA (90% CI	CFI	TLI	SRMR
1	**2.77**	0.00	0.06 (0.05–0.07)	**0.91**	0.89	**0.070**
2	**2.75**	0.00	**0.05 (0.04–0.07)**	**0.92**	**0.90**	**0.065**

Model 1 = measurement model; Model 2 = structural model; χ^2^ = chi-square; *d.f* degrees of freedom; *CFI* Comparative Fit Index; *TLI* Tucker-Lewis Index; *RMSEA* Root-Mean-Square Error of Approximation; *CI* Confidence Interval; *SRMR* Standardized Root Mean Square Residual. Figures in bold are those that meet the a priori model fitting criteria

All item loadings were significant (< 0.001) and with the expected direction. Aging, less qualifications, unemployed status and widowhood were associated with more of the ‘predisposing factors’. Of these, age had the highest factor loading (0.90). Having less household income, and higher stress positive and negative factors were associated with more of the ‘enabling’ factors. A greater number of missing teeth, higher score of PRA, greater periodontitis severity and extent, having unstable denture and having uncontrolled DM were associated with more of ‘need’ factor. The most frequent brushing and flossing, and more regular visits to the the dentist were associated with higher levels of ‘oral health behaviors’. The best indicator of evaluated ‘need’ was the missing teeth (0.78), whilst the interproximal cleaning was the best indicator in ‘oral health behaviors’ (0.73). More physical, psychological and social impacts of oral health were associated with more of the ‘perceived oral outcome’ factor.

### ABM outcomes

**Fig. 3:**
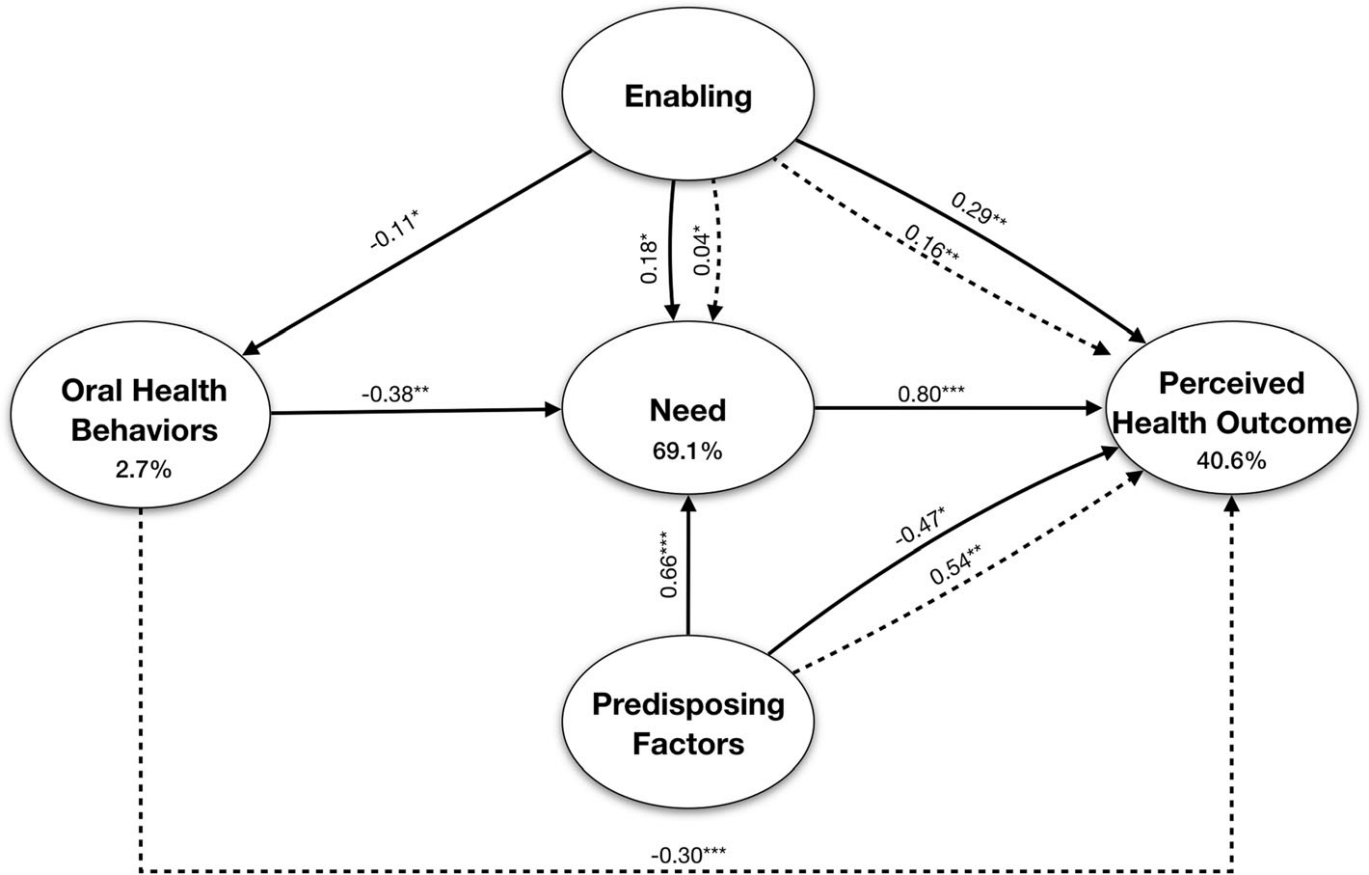
Bootstrapped ML standardized estimates for the Andersen model. *p < 0.05, ** *p* < 0.01, *** *p* < 0.001. Solid lines = direct effect; dashed lines = indirect effect

The model had acceptable fit to the data meeting all five of the latent variables (see Table 2, Model 2). Within this final model, ten paths were significant (Fig. 3), and two hypothesized paths had no significance: ‘predisposing factors’ ➔ ‘enabling’; and ‘predisposing factors’ ➔ ‘oral health behaviors’. This ABM model revealed 69.1, 2.7, and 40.6% of variance for ‘need’, ‘oral health behaviors’ and ‘perceived health outcome’, respectively (Fig. 3).

### Direct effects

Accounting for the direct effects, six of the ten pathways hypothesized in Model 2 were significant (Table 3 and Fig. 3). Less ‘predisposing factors’ (elder, less educated, be retired and widowed) was significantly linked to negative ‘perceived health outcome’ and higher ‘need’ (ß = − 0.47 *p* < 0.05, and ß = 0.66 *p* < 0.001, respectively). Greater ‘enabling’ resources was associated with higher ‘need’ (ß = 0.18 *p* < 0.05). Greater ‘oral health behaviors’ was associated with lower ‘need’ (ß = − 0.38 *p* < 0.01). A greater ‘need’ was associated with higher ‘perceived health outcome’ (ß = 0.80 *p* < 0.001). Contrary to prediction, greater ‘enabling’ resources was linked to higher ‘oral health behaviors’ (ß = − 0.11 *p* < 0.05).

**Table 3:** Direct effects for the Andersen model

Effect	ß	Bootstrap SE	Bias-corrected 95% CI	% of total effect
Predisposing Factors - Enabling	−0.01	0.06	− 0.11 / 0.09	100
Predisposing Factors - Oral Health Behaviors	−0.12	0.08	−0.26 / -0.01	_ª
Predisposing Factors - Need	0.66	0.13	0.36 / 0.77 ***	94
Predisposing Factors - Perceived Oral Outcome	−0.47	0.31	−1.01 / -0.02 *	_ª
Enabling - Need	0.18	0.01	0.04 / 0.30 *	82
Enabling - Oral Health Behaviors	−0.11	0.07	−0.22 / -0.01*	100
Enabling - Perceived Oral Outcome	0.29	0.10	0.11 / 0.42**	64
Need - Perceived Oral Outcome	0.80	0.39	0.18 / 1.40 ***	100
Oral Health Behaviors - Need	−0.38	0.08	−0.50 / -0.26 **	100
Oral Health Behaviors - Perceived Oral Outcome	0.22	0.18	−0.01 / 0.58	_ª

ß = bootstrapped standardized estimate; *SE* Standard Error; *CI* Confidence Interval

*P < 0.05, **P < 0.01

_ªCould not be calculated because of suppression effect

### Indirect effects

There were three significant indirect paths (Table 4 and Fig. 3). The path between the ‘oral health behaviors’ and ‘perceived health outcome’ was 100% indirect. In comparison, the impact of ‘enabling’ resources on evaluated ‘need’, ‘enabling’ resources on ‘perceived health outcome’, and ‘predisposing factors’ on ‘need’ were 18, 36 and 6%, respectively.

**Table 4:** Indirect effects for the Andersen model

Effect	ß	Bootstrap SE	Bias-corrected 95% CI	% of total effect
Predisposing Factors - Oral Health Behaviors	0.01	0.01	−0.01 / 0.02	_ª
Predisposing Factors - Need	0.04	0.03	−0.01 / 0.10	6
Predisposing Factors - Perceived Oral Outcome	0.54	0.31	0.07 / 1.01**	_ª
Enabling - Need	0.04	0.03	0.01 / 0.10*	18
Enabling - Perceived Oral Outcome	0.16	0.09	0.02 / 0.33**	36
Oral Health Behaviors - Perceived Oral Outcome	−0.30	0.17	−0.67/ -0.09***	100

ß = bootstrapped standardized estimate; *SE* Standard error; *CI* Confidence interval

*P < 0.05, **P < 0.01, ***P < 0.001

_ªCould not be calculated because of suppression effect

## Discussion

The results of this study confirmed our initial hypothesis, namely the number of missing teeth, PRA, the 2018 PD case definition, DM, interproximal cleaning and self-perceived stress were significant for perceived health outcome within an ABM [37]. Therefore, we highlight new factors that may be relevant in the self-perception of oral health by adult populations. Also, we observed in this population a reduced average OHQRoL (9.5 ± 11.3), though a similar decrease was previously demonstrated in a British population [38] and also worse levels in the Tromstannen - Oral Health in Northern Norway (TOHNN) study [33].

In this context, our investigation supports the notion that oral health self-perception and their factors (both direct and indirect effects) must be analyzed in a holistic way, given the existing complex interrelationships. Comprehensively, the present findings emphasize that worse levels in the “need” latent variable (periodontitis, number of missing teeth, uncontrolled DM and unstable denture) was linked to poorer perceived oral health outcomes. In other words, as an example, a participant with severe periodontitis and with a high number of missing teeth experienced worse OHRQoL. This influence on perceived oral health outcomes was very substantial (69.1%), and while for periodontitis and tooth loss our results are in agreement with previous evidence [25, 55, 56], for the remaining factors the results present novelty.

Overwhelming evidence has recognized DM as an important risk factor for PD [16, 57, 58]. In fact, our data showed a significant association between the DM status with periodontal health [57, 58]. However, DM has never been included in ABM approaches for the purpose of studying its impact on OHRQoL, and our results highlight the role of uncontrolled DM (patients with HbA1c ≥ 6.5) for these complex interactions. Hence, further studies may consider this medical condition in future investigations.

Explaining human behavior in all its complexity is a difficult task [59], and the decision-making process is influenced by social and environmental conditions [60, 61]. Onwards, our results recognize that ‘predisposing factors’ (age, education levels, marital status and occupation) have a profound direct influence on OHRQoL. Interestingly, characteristics like aging, lower levels of education, being retired or widower were directly associated with better perceived OHRQoL. Nevertheless, this association is considerably mediated by the ‘need’ latent variable, in other words, when the analysis takes into account the indirect effect of evaluated periodontal status, denture stability and DM, perception of OHRQoL by participants is affected and diminishes. This is particularly important in participants with chronic illnesses such as periodontitis because understanding and recognizing their illness is key to successful long-term periodontal maintenance and stability [62].

The majority of the elements within ABM are broadly established and overlapping [37]. Nonetheless, we added other factors into the ABM which might increase its explanatory power for OHRQoL, in particular, perceived stress into ‘enabling’ factor. Our results support an important role of perceived stress in perceived oral health outcomes. In other words, individuals with higher levels of perceived stress experienced worse OHRQoL, being in accordance with previous studies [22, 63, 64]. Furthermore, our findings suggest a negative link between ‘enabling’ factors (stress and income) and ‘oral health behaviors’. Therefore, individuals may undergo unhealthy oral behaviors (such as poor oral hygiene and avoiding dental appointments) because they might not be able to cope with stressful situations or they lack economic resources to do so, though this should be further developed in the future.

PD is an inflammatory condition caused mostly by the accumulation of polymicrobial biofilms and it is well established that periodontal health depends on the plaque control through appropriate toothbrushing techniques and careful interproximal cleaning [43, 44, 65–69]. Our results highlighted the link between oral health behaviors and periodontal status, and so, individuals with good oral hygiene and preventative measures will have better periodontal health and, consequently, better perceived OHRQoL. In the ‘oral health behaviors’ latent variable, we introduced interproximal cleaning to the ABM showing markedly impact. Our study is the first to introduce interproximal hygiene, and the results support the thesis that should be considered in future ABM studies since it strongly impacts on OHRQoL.

Although social status, economic resources, and individual health beliefs have been repeatedly profiled in an attempt to predict participant behaviors [59, 70, 71], previous efforts have focused on personal and professional bacterial removal for the treatment and control of PD [44, 65, 66]. The present study is one of the first to attempt to “unpack” likely key determinants of socioeconomic status and stress levels, personal oral health behaviors, periodontal extent, severity and inflammation, and oral health outcomes on OHRQoL and their interrelationships. We have demonstrated that OHRQoL related to periodontal status should not only consider plaque level but should undoubtedly encompass a holistic approach and consideration of all factors that may influence disease onset and extension [44, 72].

Our results indicate that four out of ten adults had some type of PD. Furthermore, almost 16 % of the adult population exhibited severe periodontitis, which is a disturbingly elevated number when compared with other European countries, that range from 6.2 to 39.9% [2, 5, 33]. On the other hand, few periodontal epidemiological surveys provided extensive and comparable information in Europe, and this is one of the first to use the new AAP/EFP consensus.

The results provided by our investigation have some notable strengths but also limitations. The cross-sectional study design applied in this study cannot identify cause and effect relationships, but rather an exploratory analysis aimed at examining the complex relationship between various contributing factors for OHRQoL. Toothbrush frequency and interproximal cleaning were self-reported items which may have introduced measurement bias. Also, HbA1c data was only available in DM patients and not to the entire population, and possibly we might have disregarded pre-diabetic patients. Another point is the low prevalence of DM (8.7%), though this prevalence is in line with recent national Portuguese evidence [73]. Additionally, OHRQoL was analysed in three different dimensions, though recent evidence suggested a four-dimensional OHRQoL mode [74] and its impact must be confirmed in future studies.

Notwithstanding, this survey has numerous strengths, including being the first study to employ ABM with a comprehensive clinical assessment of periodontal parameters as a “Need” factor, and to incorporate important variables such as diabetic status with HbA1c levels, interproximal cleaning, tooth loss, denture stability, PRA and self-perceived stress. In addition, the strengths include the representativeness and global geographic coverage based on the FHU where the study was carried out, the sample size calculation stratified for each FHU [12], the strict followed and the employment of the new AAP/EFP case definition enabling future comparability across studies [44, 75, 76].

In addition, the results validate previous findings that have evaluated items separately for periodontitis and OHRQoL [25, 77]. Thereby, including multiple items through complex statistical methods allow direct estimates, indirect estimates and information on which and how variables are related.

## Conclusion

Our findings confirm the number of missing teeth, uncontrolled diabetes mellitus, interproximal cleaning and perceived stress as important elements towards OHRQoL through ABM methodology. Periodontal Risk Assessment had low impact. Participants with a greater periodontal disease extent and severity, especially diabetic participants, have greater treatment necessity and worse OHRQoL. The number of missing teeth is highly related to increased need. Missing teeth, age, stress levels and interproximal cleaning were the items with the highest weight in their respective latent variables.

## Data Availability

Due to legal arrangements with governmental institutions, we are not authorized to disclose data.

## Abbreviations

AAP: American academy of periodontology
ABM: Andersen’s behabioral modelling
BMI: Body mass index
BoP: Bleeding on probing
CAL: Clinical attachment loss
CFA: Confirmatory factor analysis
CFI: Confirmatory fit index
CI: Confidence interval
DM: Diabetes mellitus
EFP: European federation of periodontology
FHU: Family health units
ISCED: International standard classification of education
OHIP-14: Oral health impact profile-14
OHRQoL: Oral health related quality of life
PD: Periodontal disease
PPD: Periodontal pocket depth
PRA: Periodontal risk assessment
PSS-10: Perceived stress scale-10
RMSEA: Root mean square of approximation
SEM: structural equation modelling
SoPHiAS: Study of periodontal health in almada-seixal
SRMR: Standardized root mean square residual
STROBE: Strengthening the reporting of observational studies in epidemiology
TLI: Tucker lewis index
WHO: World health organization
